# Purification and immobilization of engineered glucose dehydrogenase: a new approach to producing gluconic acid from breadwaste

**DOI:** 10.1186/s13068-020-01735-7

**Published:** 2020-06-03

**Authors:** Pinar Karagoz, Ravneet Mandair, Jinesh Cherukkattu Manayil, Jai Lad, Katie Chong, Georgios Kyriakou, Adam F. Lee, Karen Wilson, Roslyn M. Bill

**Affiliations:** 1 School of Life and Health Sciences, Aston University, Birmingham, B4 7ET UK; 2 European Bioenergy Research Institute (EBRI), Aston University, Birmingham, B4 7ET UK; 3 Department of Chemical Engineering, University of Patras, 265 04 Patras, Greece; 4 Applied Chemistry & Environmental Science, School of Science, RMIT University, Melbourne, VIC 3000 Australia

**Keywords:** Enzyme, Waste valorization, Recombinant protein, Immobilization

## Abstract

**Background:**

Platform chemicals are essential to industrial processes. Used as starting materials for the manufacture of diverse products, their cheap availability and efficient sourcing are an industrial requirement. Increasing concerns about the depletion of natural resources and growing environmental consciousness have led to a focus on the economics and ecological viability of bio-based platform chemical production. Contemporary approaches include the use of immobilized enzymes that can be harnessed to produce high-value chemicals from waste.

**Results:**

In this study, an engineered glucose dehydrogenase (GDH) was optimized for gluconic acid (GA) production. *Sulfolobus solfataricus GDH* was expressed in *Escherichia coli.* The *K*_*m*_ and *V*_max_ values for recombinant GDH were calculated as 0.87 mM and 5.91 U/mg, respectively. Recombinant GDH was immobilized on a hierarchically porous silica support (MM-SBA-15) and its activity was compared with GDH immobilized on three commercially available supports. MM-SBA-15 showed significantly higher immobilization efficiency (> 98%) than the commercial supports. After 5 cycles, GDH activity was at least 14% greater than the remaining activity on commercial supports. Glucose in bread waste hydrolysate was converted to GA by free-state and immobilized GDH. After the 10th reuse cycle on MM-SBA-15, a 22% conversion yield was observed, generating 25 gGA/gGDH. The highest GA production efficiency was 47 gGA/gGDH using free-state GDH.

**Conclusions:**

This study demonstrates the feasibility of enzymatically converting BWH to GA: immobilizing GDH on MM-SBA-15 renders the enzyme more stable and permits its multiple reuse.

## Background

The petrochemical industry continues to be an important player in the international economy [1]. In addition to fuels, petrochemical refineries produce a wide range of highly valuable platform chemicals, which are used in applications such as polymer, plastic, detergent, agrochemical and pharmaceutical production [2]. In response to concerns over the link between global warming and greenhouse gas emissions from these refineries [3], the production of platform chemicals from renewable resources has been identified as an attractive alternative to those derived from fossil fuels.

The bio-based production of commodities by fermentation is well-established [4–6]. However, the physiological limits of cellular production systems (pH, temperature, growth inhibition by a range of compounds) are a major obstacle to their cost-competitiveness [7]. Advances in recombinant protein synthesis, purification and immobilization have allowed an alternative approach, whereby purified enzymes can be used to produce metabolites [8].

Platform chemicals are essential to industrial processes. Used as crude materials for the manufacture of diverse products, their cheap availability and efficient sourcing is a focus for modern companies [9]. Currently, the majority of platform chemicals are derived from non-renewable petroleum feedstocks. In 2004, the US Department of Energy reported a list of high-value compounds that had the potential to be derived from biomass: gluconic acid (GA; C_6_H_12_O_7_) is in the top 30 high-value commodity chemicals on that list [10]. GA is a mild organic acid with an annual market volume of around 100,000 metric tonnes per year that is used in the construction, textile and pharmaceutical industries [11]. GA production via microbial fermentation processes are FDA approved and various organisms have been shown to produce this acid. *Aspergillus niger* and *Gluconobacter oxydans*, which are highly selective and efficient, have been widely used for GA production [12], but are limited to producing GA at non-toxic levels.

The hyperthermophilic archaeon, *Sulfolobus solfataricus*, typically grows at 80–85 °C, pH 2–4 and is capable of utilizing a range of carbon sources [13]. It has been recognized that *S*. *solfataricus* enzymes of the Entner–Doudoroff pathway, including glucose dehydrogenase (GDH), display substrate promiscuity and therefore offer various opportunities for potential industrial applications over a range of non-physiological temperatures [14]. Proteins produced by thermophiles also tend to be more thermostable than their mesophilic counterparts because of an increased number of hydrogen and disulphide bonds, salt bridges and hydrophobic amino acids [15]. A reduced risk of microbial contamination, decreased viscosity, higher diffusion rate and mass turnover are additional advantages of working with thermophilic enzymes at higher process temperatures [16]. One specific advantage of using GDH over glucose oxidase (GO), which produces H_2_O_2_ during the oxidation of glucose to GA [17], is that no inhibitory H_2_O_2_ is produced. However, a challenge in the widespread industrial application of any enzyme is a lack of long-term operational stability and shelf-storage life and the difficulties of recovery and re-use [18].

Immobilization of enzymes allows their separation from the product and facilitates their recovery and re-use. Immobilization has also been used by many researchers to overcome instability problems and facilitate the repetitive use of enzymes [19–21]. Furthermore, it has the possibility to improve enzymatic efficacies due to the increase of local enzyme concentration [22]. The successful development of an immobilized enzyme process depends on the properties of the enzyme, the specific immobilization process and the properties of the support, including its morphology, composition, particle size, pore structure, specific surface area, surface functional groups and rigidity [23, 24]. Due to their robust surface chemistry and tunable morphology, porous silica supports have been extensively studied for enzyme immobilization [25, 26]. Various immobilization methods, such as entrapment, encapsulation, self-immobilization, covalent bonding together with techniques to optimize the function of the enzyme once it has been immobilized have been described previously [27–29].

Over the last few years several advances in enzyme immobilization have been published. For example, cascade reactions have eliminated the need for the isolation and purification of reaction intermediates by controlling unfavourable or unstable intermediates [30]. The regeneration of expensive co-substrates, such as nicotinamide co-factors, is necessary for developing an economically viable process. For this purpose, co-immobilization of GDH with different enzymes such as xylose dehydrogenase [31], NADH oxidase [32], cyclohexanone monooxygenase [33], enoate reductase [34] or ketoreductase [35] has been investigated. The success of co-immobilization coupled with co-factor recycling increases the potential for GDH to be used in the production of a range of high-value chemicals.

The aim of this study was to synthesize recombinant GDH from *S*. *solfataricus* and immobilize the purified enzyme onto novel support materials. Two novel, hierarchically structured silica-based supports were developed for this purpose. The activity of free-state and immobilized GDH were compared under different pH and temperature conditions to assess their activity. Stability, long-term storage and the re-use of immobilized GDH were investigated. To study the potential of an enzymatic approach to waste valorization, the generation of GA from bread waste hydrolysate (BWH) using immobilized GDH was analysed.

## Results

### Purity and activity of recombinant GDH

**Fig. 1:**
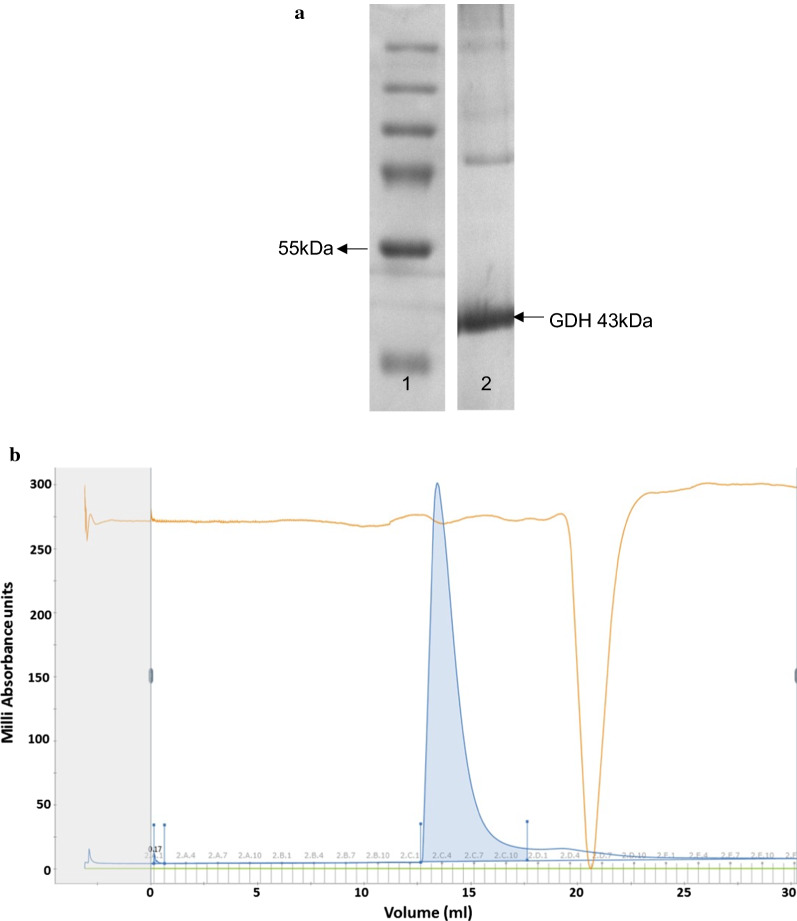
**a** SDS-PAGE analysis of purified GDH. Lane 1 shows molecular size markers, and lane 2 is GDH purified by Ni–NTA affinity chromatography. **b** Size exclusion chromatography of GDH from lane 1 in panel (**a**). The blue trace represents the UV absorbance at 280 nm and the peak is indicative of the quantity of protein detected. The brown trace reflects the conductivity monitor which measures conductivity of buffer and samples for online monitoring of the true gradient. The green trace is applied if there is another buffer being used; since this sample was run in one buffer only, the trace has a value of 0

Following affinity purification, eluted fractions containing recombinant GDH were analysed by SDS-PAGE to assess protein purity in each fraction (Fig. 1a). The purified GDH construct contained a decahistidine tag and linker and had a molecular weight of 43 kDa. The monomer could be separated via SDS-PAGE (Fig. 1a). Protein-containing fractions were analysed by BCA, pooled and subjected to size exclusion chromatography (Fig. 1b). The peak obtained indicated that GDH was 99.83% of the total sample and that the multiple-banding on the SDS-PAGE gel was an SDS-induced artefact. A single peak in the chromatograph was indicative of the homo-tetramer (Fig. 1b).

### Immobilization of purified GDH

**Fig. 2:**
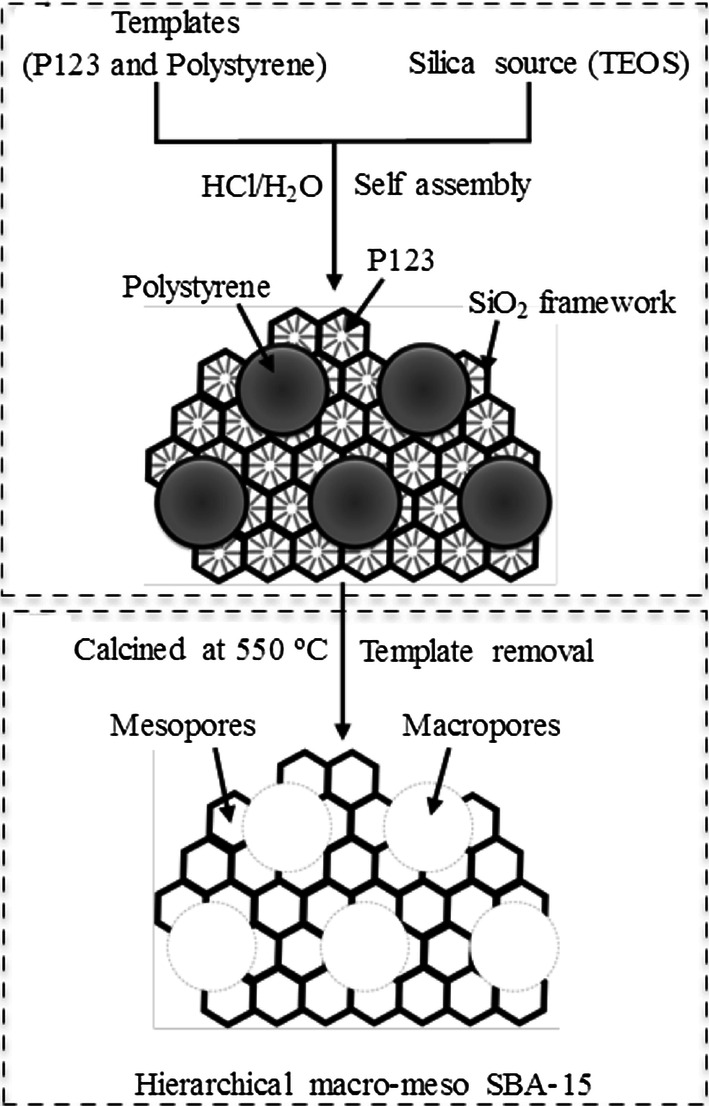
Schematic representation of the synthesis of the SiO_2_ framework containing polystyrene templates (**a**) and removal of the template (**b**) to construct the macro–meso-structured hierarchical porous material SBA-15

**Fig. 3:**
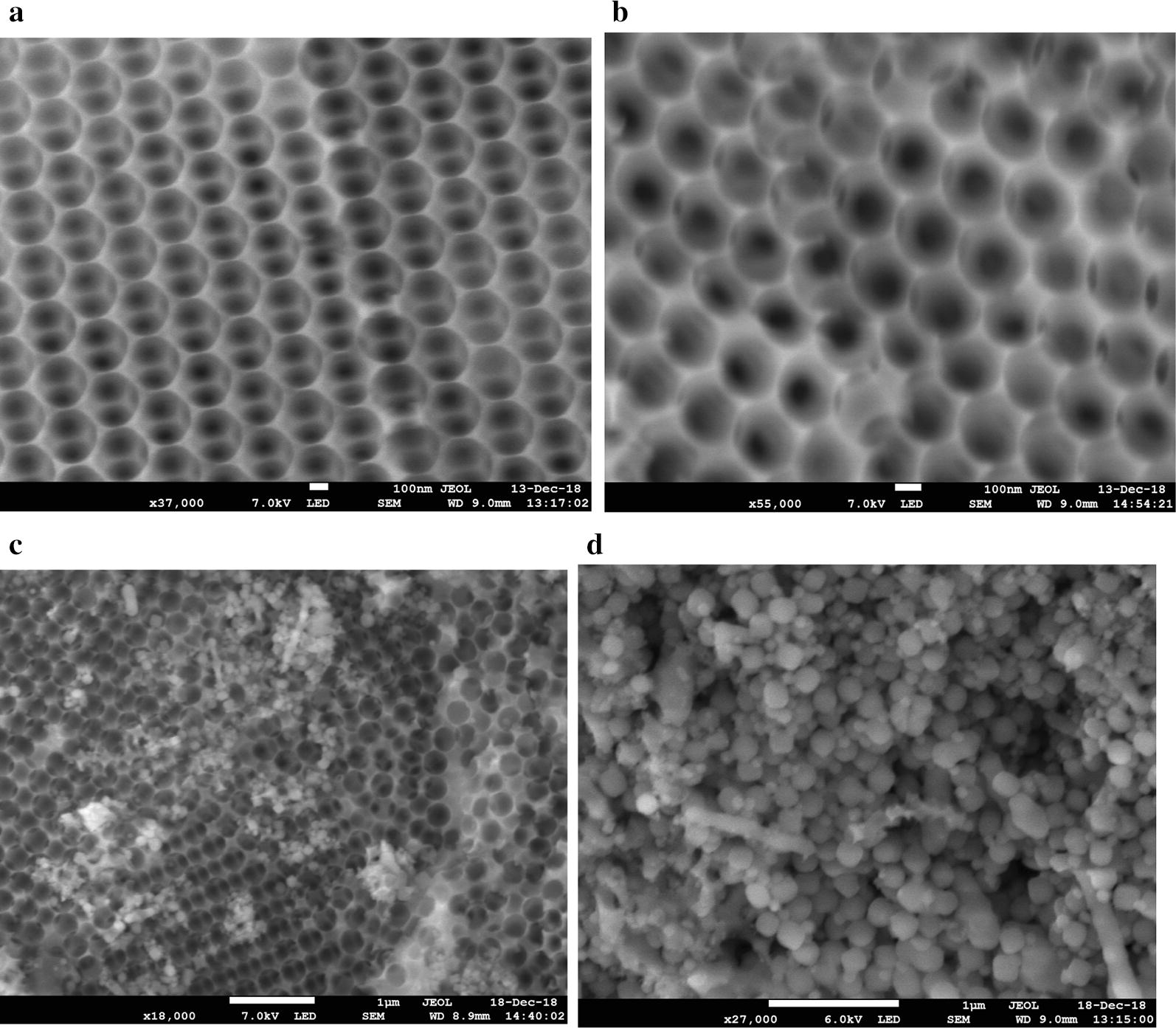
SEM of hierarchical structured **a** MM-SBA-15-300, **b** MM-SBA-15-200, **c** GDH immobilized onto MM-SBA-15-300 and **d** MM-SBA-15-200

Purified GDH was immobilized onto the different support materials listed in Table 1 without the use of cross-linking agents. The hierarchical structure of macro–meso-porous structured silica-based materials used in this study is shown schematically in Fig. 2. With the introduction of macroporosity into mesoporous SBA-15 using polystyrene beads, a well-defined interconnected macro–meso network of SBA-15 is formed. The SEM analysis confirmed the formation of macro–meso SBA-15 (Fig. 3a, b). The macropore diameter was consistent with the size of the polystyrene bead used in the synthesis. Mesoporous generation was further confirmed by N_2_ porosimetry and both MM-SBA-15 samples possessed surface areas of 270-380 m^2^/g. The values were comparable with the commercial macroporous polymer support, ECR8309F, which has a slightly lower surface area of 70–220 m^2^/g. The other commercial supports have significantly higher (750–850 m^2^/g, ECR1090M) or lower (80–120 m^2^/g, ECR1030M) surface areas (Table 1). The pore diameters of macro–meso-porous structured MM-SBA-15-300 and MM-SBA-15-200 were 2 to 10 times higher than commercial supports (ECR8309F, ECR1090M, ECR1030M). In addition, the large surface areas of MM-SBA-15-300 and MM-SBA15-200 are comparable with those of commercial supports. Under the same immobilization conditions, 3 mg purified GDH was immobilized onto 1 g support. Table 1 shows that the immobilization efficiency of macro–meso-porous structured silica-based MM-SBA-15-300 and MM-SBA-15-200 were significantly higher than that of all three-commercial support materials. 98% of the GDH was successfully immobilized onto MM-SBA-15 supports. SEM of immobilized GDH onto these hierarchical silica supports is shown in Fig. 3.

**Table 1:** Specification of support materials and their immobilization efficiencies

Support Material	Type of support	Surface functional group	Immobilization type	Particle size (µm)	Surface area (m^2^/g)	Pore diameter (nm)	Immobilization efficiency (%)
ECR8309F	Amino C2 methacrylate	NH_2_ (short spacer)	Covalent or ionic	150–300	70–220	60–120	59 ± 10
ECR1090M	Macroporous styrene	None	Adsorption/hydrophobic interaction	300–700	750–850	90–110	76 ± 16
ECR1030M	DVB/Methacrylate	None	Adsorption/hydrophobic interaction	300–700	80–120	20–30	73 ± 11
MM-SBA-15-300	Hierarchical porous silica	None	Adsorption/entrapment	100–300^a^	350–380	290–300 (4.4^b^)	98 ± 2
MM-SBA-15-200	Hierarchical porous silica	None	Adsorption/entrapment	100–300^a^	270–300	240–250 (3.9^b^)	98 ± 2

^a^Particle size determined by SEM analysis

^b^Mesopores determined from N_2_ porosimetry by BJH analysis

### Thermostability of free-state and immobilized GDH

**Fig. 4:**
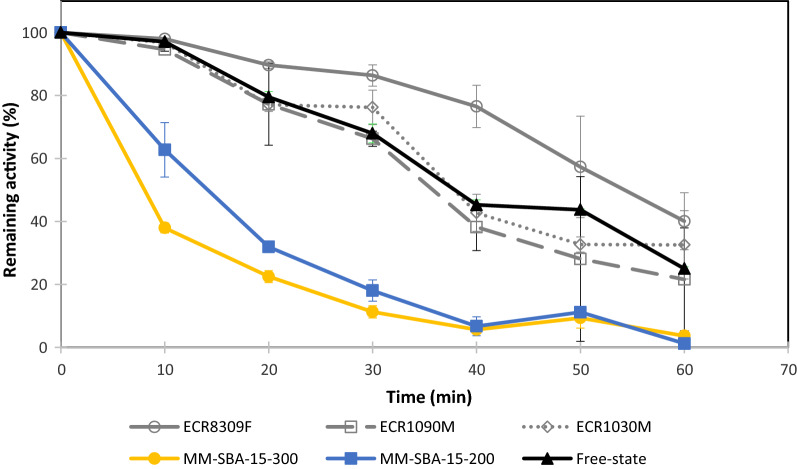
Thermostability of free-state and immobilized GDH on commercial (ECR8309F, ECR1090M, ECR1030M) and hierarchical (MM-SBA-15-300 and MM-SBA-15-200) support materials at 80 °C

To understand the stability of GDH under non-physiological conditions, free-state and immobilized GDH were assayed at 55, 65, and 80 °C over 18 h. The free-state and immobilized GDH were thermostable at 55 and 65 °C, since no significant activity loss was detected (data not shown). The specific activity of free-state GDH at 55 °C in 100 mM glucose was calculated to be 2.354 µmol/min/mg (based on a 3-h reaction duration). The specific activities of immobilized GDH on ECR8309F, ECR1090M, MM-SBA-15-300 or MM-SBA-15-200 were 1.071, 1.158, 1.422, 2.305 or 2.345 µmol/min/mg (stdev < 0.03), respectively. These values were set to 100% activity and activity loss was reported relative to these initial values. However, the samples heated at 80 °C for 18 h lost all activity. Figure 4 shows that the thermostability of GDH immobilized onto ECR8309F, an amino methacrylate support, was significantly higher than the stability of free-state GDH over 60 min. However, the thermostability of GDH immobilized onto ECR1090M or ECR1030M was not significantly increased. The thermostability of GDH immobilized onto macro–meso-structured MM-SBA-15 supports was significantly lower than GDH immobilized onto commercial supports.

### Re-usability of immobilized GDH

**Fig. 5:**
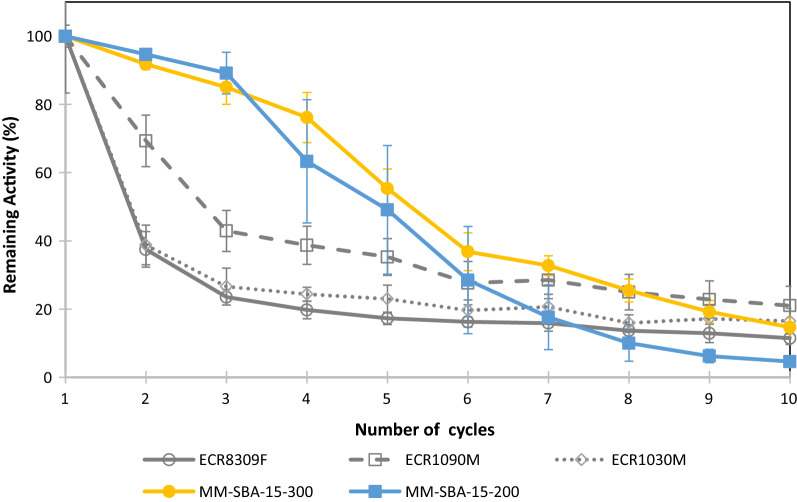
Re-usability of immobilized enzyme on commercial and hierarchical supports and their loss of activity after each re-use

In this study, the re-usability of purified GDH was studied over 10 cycles. As described above, activity loss was reported relative to initial values. During the first 5 cycles, the activity of GDH immobilized on macro–meso-structured MM-SBA15-300 and MM-SBA15-200 was significantly more stable than GDH immobilized on commercial supports (Fig. 5). At the end of the 5th cycle, GDH immobilized on MM-SBA-15-300 and MM-SBA-15-200 retained 55% and 49% of its initial activity, respectively. The activity of GDH immobilized on ECR1090M, ECR1030M and ECR8309M were 35, 22, and 17% of initial activity, respectively.

### Storage stability of immobilized GDH

**Fig. 6:**
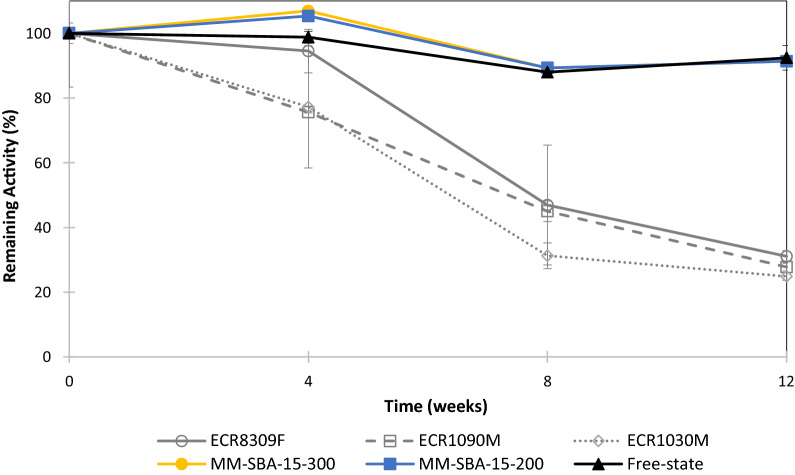
Effect of the immobilization and the nature of the support material on the activity of immobilized GDH stored at 4 °C for 12 weeks

To determine storage stability, free-state and immobilized GDH were stored at 4 °C for 12 weeks and the enzymatic activity was measured at various time points. Figure 6 shows that the catalytic properties of free-state GDH were well preserved at 4 °C, with 90% activity being retained at the end of the 12-week storage period. However, the activity of GDH immobilized on commercial supports was significantly reduced with increasing storage time. The amount of enzyme leaking from the supports increased with time. After 8 weeks, 24.7–36.3% of the initial protein loading had leaked from the supports and at the end of the 12-week storage period, the amount of leaked protein increased to 30–43% (Additional file 1: Table S1). At the end of the 12-week storage period, the remaining activity of GDH immobilized onto MM-SBA-15 supports was 60% higher than that of GDH immobilized onto commercial support materials. In addition, during the 12-week storage period, the remaining activity of GDH immobilized onto silica supports showed similar characteristics to the free-state enzyme.

### Enzymatic activity of immobilized GDH

The Michaelis–Menten model was used to calculate the apparent *V*_max_ and *K*_*m*_ values of free-state GDH and GDH immobilized onto MM-SBA-15 supports (Table 2). The apparent *V*_max_ and *K*_*m*_ values of free-state GDH were 5.91 U/mg and 0.87 mM, respectively. After immobilization, the *V*_max_ values of the GDH immobilized onto MM-SBA-15-300 and MM-SBA-15-200 reduced to 0.83 and 1.14 U/mg, respectively. Overall, when GDH was immobilized onto hierarchical supports, there was a five to sevenfold decrease in *V*_max_. Compared with free-state GDH, the *K*_*m*_ values of GDH immobilized onto MM-SBA-15-300 and MM-SBA-15-200 increased from 0.87 to 1.88 and 1.22 mM, respectively. Compared to the free-state enzyme and the enzyme immobilized onto MM-SBA-15-200, higher *K*_*m*_ values were observed for GDH immobilized onto MM-SBA-15-300.

**Table 2:** Kinetic parameters for free-state and immobilized GDH

Kinetic parameters	Free-state	MM-SBA-15-300	MM-SBA-15-200
*V*_max_ (U/mg)	5.91	0.83	1.14
*K*_*m*_ (mM)	0.87	1.88	1.22
*k*_cat_ (1/s)	3.94	0.55	0.76

### Optimum conditions for GDH immobilized on macro–meso-structured SBA supports

**Fig. 7:**
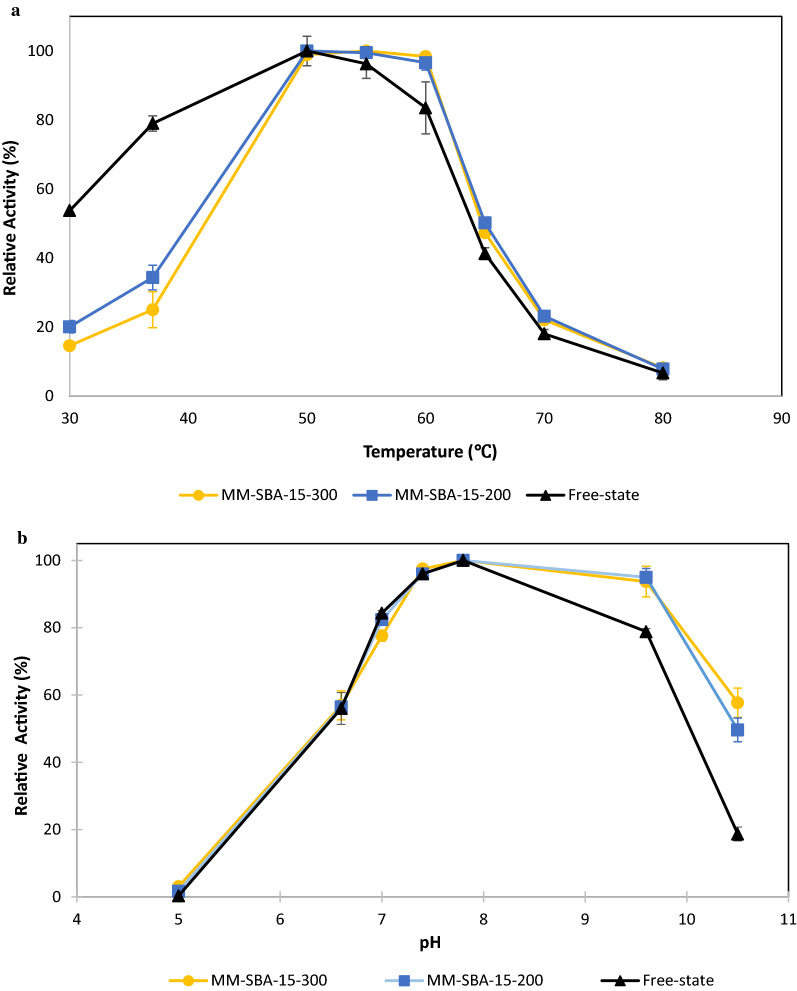
Effect of **a** temperature and **b** pH on the activity of GDH when it is in a free-state or immobilized on MM-SBA-15-300 or MM-SBA-15-200

The activity of GDH immobilized onto MM-SBA-15-300 and MM-SBA-15-200 was investigated at different temperature and pH conditions. The effect of temperature on the activity of free-state and immobilized GDH was investigated from 30 to 80 °C at pH 7.4. Free and immobilized enzyme showed optimum activity at 50–55 °C (Fig. 7a). At temperatures higher than 50 °C, immobilized GDH showed better relative activity than the free-state enzyme. As shown in Fig. 7a, GDH immobilized on MM-SBA-15-300 and MM-SBA-15-200 showed similar characteristics at all temperatures. Figure 7b shows the effect of pH on the activity of free and immobilized GDH. The optimum pH for immobilized and free-state GDH was pH 7.8. At pH 7.8, the specific activities of free-state GDH, immobilized GDH on MM-SBA-15-300 and MM-SBA-15-200 were calculated to be 25.11, 24.29, 24.08 µmol/min/mg, respectively. Even at high pH conditions, the activity of GDH was retained, with higher activity remaining for immobilized GDH. At pH 9.6, the residual activity of free-state GDH was 78.8%, while the activity of GDH immobilized onto MM-SBA-15-300 and MM-SBA-15-200 was 93.7% and 95.0, respectively. When the pH was adjusted to 10.5, immobilized GDH retained half its activity, while the free-state GDH retained only 18.7% of its activity.

The effect of enzyme/support ratio on GDH immobilization onto macro–meso-structured silica supports was investigated by increasing the amount of protein during the immobilization process. Table 2 shows the immobilization efficiency and relative activity of the immobilized GDH at different enzyme loadings. Limitations in the yield of purified enzyme, meant that it was not possible to test the materials at GDH/support ratios higher than 25/1, mg/g. As shown in Table 3, almost 100% of the GDH was successfully immobilized when the GDH/support ratio was between 1/1 and 5/1. After this point, immobilization efficiency slightly decreased to 98% and 82% for MM-SBA-15-300 and MM-SBA-15-200, respectively. However, the relative activity of the immobilized GDH increased until the GDH/support ratio reached 5/1. When MM-SBA-15-300 was used as a support, suitable GDH/support ratios were between 3/1 and 25/1, which achieved high relative activity and high immobilization efficiency. Similarly, for MM-SBA-15-200, suitable GDH/support ratios were between 3/1 and 10/1.

**Table 3:** Effect of enzyme/support ratio on enzyme immobilization efficiency and activity

GDH/support ratio (mg/g)	1/1	2/1	3/1	5/1	10/1	25/1
Immobilization efficiency (%)						
MM-SBA-15-300	100 ± 0	100 ± 0	99 ± 1	96 ± 4	98 ± 1	98 ± 1
MM-SBA-15-200	100 ± 0	100 ± 0	99 ± 1	100 ± 0	98 ± 1	82 ± 2
Relative activity (%)						
MM-SBA-15-300	89 ± 7	91 ± 3	93 ± 1	95 ± 1	98 ± 1	99 ± 1
MM-SBA-15-200	64 ± 4	86 ± 5	98 ± 2	99 ± 1	99 ± 1	99 ± 1

### Conversion of BWH

**Fig. 8:**
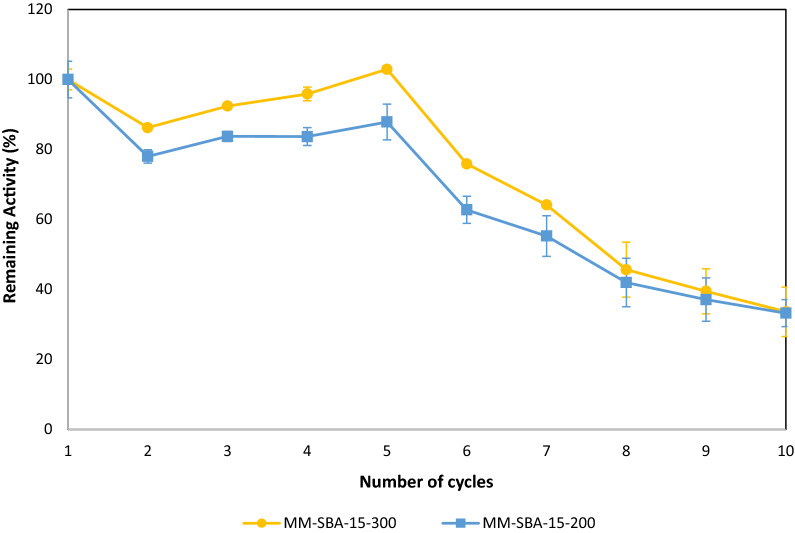
Re-usability of GDH immobilized on MM-SBA-15-300 or MM-SBA-15-200 in BWH and the loss of activity after each re-use cycle

**Fig. 9:**
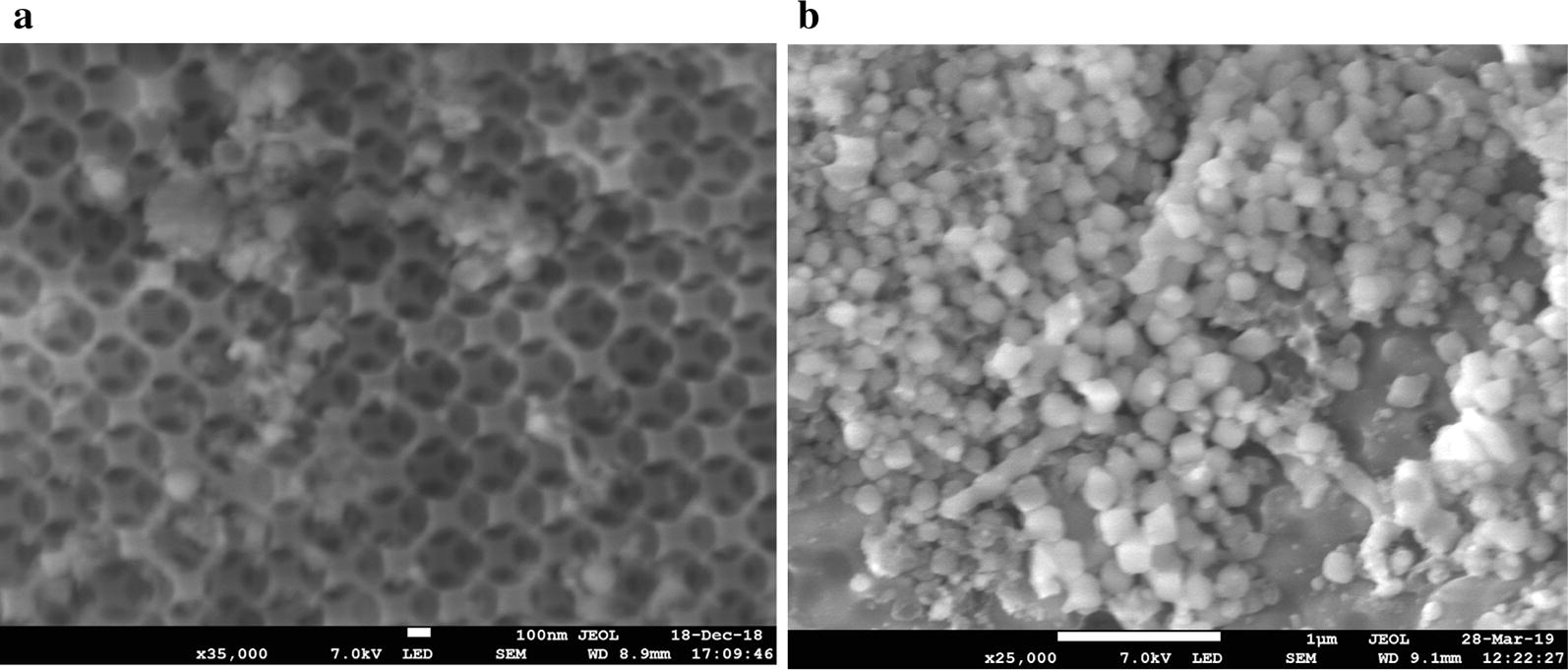
SEM of immobilized GDH on **a** MM-SBA-15-300 or **b** MM-SBA-15-200 after 10 cycles

To investigate the stability and activity of free-state and immobilized GDH on waste-based sugars, BWH was used. BWH was prepared as described previously and the glucose concentration of BWH was 63.1 ± 3.3 g/L. This sugar-rich liquor was used as the sole glucose source in the reaction mixture. The final glucose concentration was adjusted to 100 mM using BWH. Figure 8 shows the remaining activity after each use of immobilized GDH and Fig. 9 shows the SEM images of the immobilized supports after the 10th cycle. After 7 cycles, the remaining activities of immobilized GDH were 64 and 55% on MM-SBA-15-300 and MM-SBA-15-200, respectively. After this point, further use of immobilized enzymes reduced the activity to more than 50% of its initial activity. After 10 cycles, the remaining activities of GDH immobilized onto MM-SBA-15-300 and MM-SBA-15-200 were reduced to 34 and 33%, respectively. As can be seen from Table 4, 45% of the glucose in BWH was converted by free-state GDH. A twofold decrease in the conversion yield was observed when immobilized GDH was used. GA production efficiencies by free-state GDH and GDH immobilized onto MM-SBA-15-300 and MM-SBA-15-200 were calculated as 47, 32 and 35 gGA/gGDH, respectively. In every cycle, the GA production efficiency of the GDH immobilized onto MM-SBA-15-200 was higher than MM-SBA-15-300 and after the 10th cycle, the GA production efficiency of GDH immobilized on MM-SBA-15-200 was double that of MM-SBA-15-300 (Fig. 9).

**Table 4:** Biocatalytic productivity of immobilized and free-state GDH on bread waste hydrolysate (BWH)

Parameter	Free-state GDH	GDH-MM-SBA-15-300 1st cycle	GDH-MM-SBA-15-200 1st cycle	GDH-MM-SBA-15-300 5th cycle	GDH-MM-SBA-15-200 5th cycle	GDH-MM-SBA-15-300 10th cycle	GDH-MM-SBA-15-200 10th cycle
Conversion of glucose in BWH (%)	45 ± 8	23 ± 2	25 ± 2	21 ± 4	23 ± 3	25 ± 2	22 ± 2
Gluconic acid (GA) production from BWH (gGA/gGDH)	47 ± 1	32 ± 1	35 ± 1	31 ± 1	38 ± 2	12 ± 1	25 ± 3

## Discussion

Effective enzyme immobilization is known to be critically dependent on the choice of the support material [20] and the purity of the enzyme being immobilized: the presence of other, contaminating proteins in an enzyme preparation reduces both enzyme loading and the apparent biocatalytic performance of the enzyme. The high purity of the GDH preparation (99.83%) achieved in this study is therefore a substantial benefit to further studies. Previous studies showed that the GDH monomer from *S*. *solfataricus* is composed of 366 amino acid residues (with a molecular weight of 41 kDa) and forms tetrameric assemblies in solution [14]. In addition, the crystal structure of *S*. *solfataricus* GDH confirmed the enzyme to be a homotetramer [36]. Our SDS-PAGE gel analysis and size exclusion chromatography data for the GDH purified in this study are consistent with this.

Due to the presence of hydroxyl groups that facilitate enzyme binding, silica-based materials are efficient and attractive supports for enzyme immobilization [31, 37] and were therefore the subject of this study. The surface area and pore diameter of an immobilization support determines its loading capacity and the size of protein that can be immobilized. Small pore sizes may limit diffusion and increase the risk of pore blockage. Furthermore, immobilization can only be achieved on the external surface of a support if the pore size is too small [38]; desorption is also more likely to occur as the operating temperature is increased [39]. It has therefore been suggested that the pore diameter should be at least four to fivefold larger than the equivalent dimension of the enzyme to be immobilized, but not so large such that it affects the mechanical properties of the support material [20]. In this study, the immobilization efficiency of GDH was significantly higher on our novel MM-SBA-15 silica support materials with macro–meso-structured hierarchical pores than on commercially available silica supports. The hierarchical supports used in this study have no functional groups on their surface, with only silanols having the potential to affect enzyme immobilization. However, the enzyme immobilization yield of the nanoporous silica used to synthesize the hierarchical supports in this study was significantly lower than MM-SBA-15 supports (Additional file 1). As a result of its relatively small macro-pore diameter, and consistent with the observations above, more immobilized enzyme was visible on the surface of MM-SBA-15-200 than MM-SBA-15-300. The success of the immobilization is a result of entrapment of the enzyme inside the macro–meso-structured hierarchical pores of MM-SBA-15. Similar behaviour was reported when commercial GDH and XDH enzymes were co-immobilized onto hexagonal mesoporous SBA 15 [37].

The activity of an enzyme under different physicochemical conditions is a function of its stability [40]. The prevention of subunit dissociation of multimeric enzymes, such as GDH, is an important consideration in this respect [41]. Immobilization (both physical and chemical) has been shown to be an effective strategy to address this problem [42], with previous studies demonstrating that immobilization improves the stability of multimeric enzymes [27, 36]. These findings suggest that our hierarchical silica supports may have played an important role in the prevention of subunit dissociation of the tetrameric GDH used in this study.

Immobilization often increases enzyme thermostability [43–45]. The chemistry of a support material, including the nature of its functional groups and the length of the matrix-enzyme spacer all affect thermostability [46]. In this study, we compared the thermostability of immobilized GDH on commercial supports and our novel hierarchically structured MM-SBA-15. We carried out thermal shocks on immobilized and free-state GDH at 55, 65, and 80 °C overnight; no significant activity loss was detected at 55 and 65 °C. However, when the temperature was increased to 80 °C, all immobilized and free-state GDH lost activity. To understand the robustness of immobilized GDH on different supports, heat shocks at 80 °C were carried out for shorter periods. Compared to free-state GDH, the thermostability of GDH immobilized on ECR8309F was significantly improved. The activity of immobilized GDH on other commercial supports had similar activities to free-state GDH. Unexpectedly, GDH immobilized on MM-SBA-15 showed reduced thermostability. This may be caused by the thermal conductance properties of silica. Similar observations have been reported in previous studies using silica-based support materials at very high temperatures [47].

Free-state enzymes are difficult to recover and re-use [34], while immobilization of enzymes allows their cost-effective re-use in repeated batch or continuously running processes [48, 49]. In this study, immobilized GDH was used 10 times. At the end of the 5th cycle, GDH immobilized on commercial supports had lost more than 65% of its initial activity. However, GDH immobilized on MM-SBA-15-300 and MM-SBA-15-200 had lost 41% and 51% of its initial activity, respectively. The reduction of enzymatic activity after each cycle is likely caused by the leakage of unbound enzyme and/or accumulated reaction product on the surface of the material, thereby limiting the diffusion of the substrate and the product. In previous studies, commercial GDH immobilized on nanoSiO_2_ and SBA-15 retained 50 and 43% of its initial activity, respectively [31], while glucose-6-phosphate dehydrogenase (G6PD) immobilized on silanized silicon retained 38% of its initial activity after 6 cycles [50]. Improved storage stability of immobilized enzymes is another advantage over the corresponding free-state preparations [48]. The activity of GDH immobilized on commercial supports was significantly reduced over time compared with GDH immobilized on MM-SBA-15.

Despite the reduced thermostability of GDH on MM-SBA-15 supports at 80 °C, our data demonstrated substantial enzyme activity at normal working temperatures, long-term enzyme re-usability and improved storability. To further improve the activity of GDH immobilized on MM-SBA-15, the effects of temperature and pH on the activity of free and immobilized GDH were tested. We noted that immobilization on MM-SBA-15 did not shift the optimum working temperature of GDH. The activity of the free-state enzyme was slightly decreased when the temperature increased from 50 to 60 °C, while the activity of the immobilized enzyme was not affected. The optimum pH of an immobilized enzyme depends upon the particular enzyme and the chemistry of the support [51]. In this study, we tested the activity of GDH over a range of pH. At pH greater than 7.5, the activity of immobilized GDH on MM-SBA-15 supports was significantly higher than free-state GDH. Usefully, at high pH, above the pK_a_ of GA, GA can be separated from the reaction mixture by simple separation methods, such as membrane filtration [31].

Specific area determines the loading capacity of a support and the ratio of enzyme to support material is a crucial immobilization parameter for achieving high efficiency and high reaction rates [24]. Immobilizing high concentrations of enzyme is preferable from an economical point of view [20], but increasing enzyme concentrations on the support material can also increase the cost of the process [52]. Furthermore, excessive loading may have an effect on enzyme leakage, pore blockage and mass transfer between liquid and solid [51]. Hence, working at a reasonable enzyme/support ratio is important in developing catalytic bioprocesses. In this study, we used different enzyme/support ratios and quantified immobilization efficiencies and GDH activity. At enzyme loading ratios between 1/1 and 10/1 (mg/g), almost all of the GDH was immobilized onto the MM-SBA-15 supports. However, enzyme activity was reduced at decreased enzyme loading ratios, which might be a result of diffusional constraints. The highest GDH/support ratio tested in this study was 25/1 (mg/g) where more than 80% of the enzyme was successfully immobilized on MM-SBA-15-200. Under the same conditions, 98% of the enzyme was immobilized on MM-SBA-15-300, probably due to its higher surface area and pore diameter.

The kinetic parameters of free-state GDH and GDH immobilized on MM-SBA-15 supports showed that immobilization leads to drop in the *V*_max_. This expected result might be caused by mass transfer limitations [24]. As a result of enzyme attachment, some of the active sites of GDH could be blocked, which would reduce the reaction rate and *V*_max_ [31]. Similar observations have been made when GDH from *Bacillus megaterium* was immobilized on DEAE-Sephadex [53]. Diffusional limitations caused by immobilization also lead to an increase in *K*_*m*_ values for GDH immobilized on MM-SBA-15 supports. As shown in Fig. 3, while GDH was mainly immobilized on the surface of MM-SBA200, GDH was predominantly immobilized inside of the pores of MM-SBA-15-300. Similarly, after immobilization of commercial GDH onto silica supports, depending of the pore structure of the supports, a reduction in *V*_max_ of up to 1.87-fold, and an increase in *K*_*m*_ of up to 1.78-fold were reported [31].

Increasing generation rates and quantities of food waste are a global concern [54, 55]. Bread waste contains high amounts of carbohydrate that can be used as a potential feedstock in bioprocesses for the generation of various biobased products, replacing those from fossil resources with their associated economic and environmental concerns [56]. In this study, we converted bread waste to GA, a platform chemical, by using free-state and immobilized GDH. We re-used immobilized GDH 10 times in reaction mixtures containing BWH as a substrate. After the 7th cycle, the activity of GDH immobilized on MM-SBA-15 was above 50% of its initial activity. The GA yield from GDH immobilized on MM-SBA-15-300 was higher than that on MM-SBA-15-200, in each cycle. Further investigations such as the effect of substrate levels and BWH/enzyme loading ratios on the GA production yield are needed. To develop a more effective and economic bioprocess, GDH could be coupled with an additional enzyme for co-factor recycling. In a previous study, commercial GDH was successfully co-immobilized with xylose dehydrogenase (XDH) on a silica surface for co-factor recycling [31]. In another study, commercial GDH and NADH oxidase (NOD) enzymes were immobilized on aldehyde functional ReSyn polymer microspheres for co-factor recycling [32]. In this study, we investigated an engineered GDH and the potential of hierarchical porous materials for biocatalytic applications. Co-enzyme recycling as a part of multi-enzyme cascades could be an attractive route for green chemical production from waste. However, the main focus of this study is to investigate the potential of hierarchical supports and the activity of engineered GDH when immobilized upon them. Our results show the long-term stability and the activity of engineered GDH on MM-SBA-15 supports. Potential future applications could include its coupling with other enzymes for co-factor recycling.

## Conclusion

Recombinant GDH from *S*. *solfataricus* was successfully purified and immobilized onto different supports. GDH immobilized onto a novel, hierarchically structured macroporous–mesoporous silica support showed great potential for GA production from glucose as well as BWH. Hierarchically porous MM-SBA-15 showed better immobilization efficiency than commercial supports. Moreover, GDH immobilized onto these materials showed excellent reusability.

The use of GDH immobilized onto hierarchically structured supports has the potential to be incorporated into next-generation bio-refineries and waste valorization studies. GA, the product of the enzymatic process developed in this study, can be used for industrial purposes or can be converted to other high-value platform chemicals such as malic acid. Co-immobilization of the GDH produced in this study with related enzymes to recycle the co-factor, NAD^+^, may help develop attractive processes for more efficient platform chemical production.

## Materials and methods

### Microorganisms and enzyme production

The *GDH* gene from *S. solfataricus* was cloned into the expression vector pET30a using *Nde*I and *Bam*HI as the 5ˊ and 3ˊ restriction sites, respectively. The full-length gene was synthesized by GenScript with sequence optimized for expression in *E. coli* and the vector was transformed into BL21-DE3 competent cells. Cells were grown in LB media supplemented with 50 µg/mL kanamycin and grown at 37 °C and 220 rpm to *A*_600_ = 0.6. 1 mM (final concentration) IPTG was then added and the cells were grown at room temperature for 18 h at 220 rpm. Cells pellets were collected by centrifugation and re-suspended in a solution containing 100 mM HEPES pH 7.4, 200 mM MgCl_2_, 10% glycerol. Cell lysates were prepared using a cell disruptor system (Bandelin, SONOPULS) and heat treated at 70 °C. Cellular debris was removed by centrifugation and the crude lysate was bound overnight to Ni–NTA resin (Qiagen) and eluted with buffer containing 600 mM imidazole, 100 mM HEPES pH 7.4, 200 mM MgCl_2_, 10% glycerol. The protein was then dialysed into 100 mM HEPES pH 7.4, 200 mM MgCl_2_, 10% glycerol. Size exclusion chromatography was carried out on an ÄKTA Pure chromatography system (GE) using a Superdex™ Increase 10× 300GL column. Fractions containing GDH were pooled and their protein concentration determined using the bicinchoninic acid assay (BCA; Reagent Compatible BCA Assay Kit, ThermoFisher Scientific) and a Nanodrop device (ThermoFisher). Samples were snap frozen in liquid nitrogen and stored at − 80 °C.

### GDH activity assay

The activity of GDH was assayed in 100 mM HEPES pH 7.4, 2.5% glycerol, 30 mM MgCl_2_, 100 mM glucose, 5 mM NAD^+^. Unless otherwise stated, the activity assay for both immobilized and free-state GDH was conducted in a water bath at 55 °C for 3 h. At the end of the assay, samples were placed into pre-cooled racks and supernatants were filtered through 0.2-μm filters. The activity of GDH was detected by measuring the NADH concentrations of the supernatants with a plate reader at 340 nm. The enzyme concentration was measured using the BCA method.

### Determination of kinetic constants

The kinetic constants for free-state and immobilized GDH were determined by measuring the reaction rates at regular time intervals and different substrate levels (0, 0.1, 0.5, 1, 2.5, 5, 10, 25, 50, 100 mM). *K*_*m*_ and *V*_max_ values were calculated from Lineweaver–Burk plots.

### Support materials

To immobilize GDH, three different commercial enzyme supports; methacrylate-based ECR8309F, styrene-based ECR1090M and methacrylate/divinylbenzene-based ECR1030M were purchased from Purolite, UK. Meso-macroporous SBA-15 (MM-SBA-15) was synthesized via a modified true liquid crystal templated (TLCT) SBA-15 synthesis [57] which included a hard macropore template of polystyrene spheres. Polystyrene spheres were synthesized as reported elsewhere [58]. The structure-directing template (Pluronic P123l; 2 mL) was mixed with hydrochloric acid acidified water (~ pH 2, 2.1 mL) and sonicated at 40 °C to produce a homogeneous gel. The sol–gel exhibited a hexagonal mesophase. 6 g polystyrene were added to the sol–gel with stirring, resulting in the formation of a viscous solution. Tetramethoxysilane (4 mL) was added and mixed with stirring to form a homogeneous liquid at 40 °C. The evolved methanol was removed under a light vacuum (0.12 bar) at 40 °C to form a viscous gel overnight. The gel was exposed to the atmosphere at room temperature for 24 h to complete condensation before calcination at 500 °C for 6 h in air (ramp rate 2 °C/min). The products were designated MM-SBA-15-300 and MM-SBA-15-200, 300 and 200 according to the size of the polystyrene beads used in the synthesis. Low-angle XRD patterns were recorded on a Bruker D8 Advance diffractometer fitted with an X’celerator detector and Cu Kα (1.54 Å) source over the range 2*θ* = 0.3–10°. Nitrogen porosimetry was measured on a Quantachrome Nova 4000 porosimeter and analysed with NovaWin software. Samples were degassed at 120 °C overnight prior to analysis at − 196 °C. Brunauer–Emmett–Teller (BET) surface areas were calculated in the relative pressure range of 0.01–0.2. The Barrett–Joyner–Halenda (BJH) method was used to calculate pore diameters and pore volumes in the desorption isotherm for relative pressures > 0.35. The polystyrene bead size and morphology of synthesized MM-SBA-15-200 and MM-SBA-15-300 were evidenced using scanning electron microscope (SEM). The characteristics of all support materials used in this study are shown in Table 1.

### Immobilization of GDH

The immobilization of recombinant GDH onto different support materials was done by gently mixing the enzyme and the support material with enzyme support solution (ES; 100 mM HEPES pH 7.4, 2.5% glycerol, 50 mM MgCl_2_) at room temperature for 10 min. Unless otherwise stated, 0.03 mg GDH and 0.01 g support material were mixed with 1 mL ES. Following this, mixtures were stored at 4 °C overnight. The amount of immobilized enzyme was detected by measuring the protein concentration before and after immobilization. Immobilization yield (%) was calculated using the equation bellow:

$${\text{Immobilization yield }}\left( \% \right) = \frac{{C_{0} - C_{s} }}{{C_{0} }} \times 100,$$

where *C*_0_ and *C*_*S*_ denote the amount of enzyme before and after immobilization (mg), respectively.

### Thermal stability of free-state and immobilized GDH

The thermal stability of free-state and immobilized GDH was investigated at 55, 65 and 80 °C after 1, 3, 18 and 24 h. 0.03 mg free-state or immobilized GDH in 500 μL ES were incubated as required, instantly cooled down on ice for 2 min and stored at 4 °C for 20 min. Cooled samples were gently mixed with assay mixture and the activities of free and immobilized GDH were evaluated as described above.

### Reusability of immobilized GDH

To detect the reusability of GDH immobilized onto different supports, the immobilized enzyme was washed with ES after each use and stored at 4 °C overnight and then re-suspended in fresh assay mixture to determine remaining activity. This procedure was repeated 10 times. The activity of the enzyme in the first cycle was defined as 100% and the remaining activities were calculated as follows:

$${\text{Remaining activity}} \left( \% \right) = \frac{{a_{{x_{2 \ldots 10} }} }}{{a_{1} }} \times 100,$$

where *a*_1_ and *a*_*X*2….10_ denote the enzyme activity at the end of the first and subsequent cycles, respectively.

### Effect of immobilization on storage stability

The free-state and immobilized GDH were stored at 4 °C to detect the effect of the support material and immobilization on their storage stability. The activity of the GDH was determined at 4-week time intervals.

### Determination of optimum temperature and pH

The activity profiles were studied at different temperatures (30–80 °C) and pH ranges (5.0–10.5). The pH of the GDH assay buffer was adjusted using 3 M HCl, 1 M and 100 mM NaOH.

### Preparation of bread waste hydrolysate (BWH)

Sliced, soft white packaged bread (Warburton) was used as a feedstock. According to the manufacturer’s nutritional information, one slice of bread (wet weight 28.8 g) contained 13.1 g carbohydrate (of which 0.8 g were sugars), 2.6 g protein and 0.6 g fat. Slices were dried at 37 °C overnight and broken into small pieces by hand. Pieces from 1 slice of bread were transferred into 250-mL Erlenmeyer flasks, 100 mL water was added and the flask was placed in a shaking incubator at 60 °C, 200 rpm for 1 h. As previously described [59], 5 mg α-amylase from *Aspergillus oryzae* (Cat. No. 10065, Sigma) and 7 mg amyloglucosidase from *Rhizopus* sp. (Cat. No. A9228, Sigma) were added to each flask and incubated at 60 °C for 1.5 h. Solid particles were separated by centrifugation for 20 min, at 4100×*g* and 4 °C. The supernatant was transferred into a flask and autoclaved at 121 °C for 15 min. The glucose concentration of the hydrolysate and utilization of glucose by GDH was measured using glucose assay (Cat. No. GAGO20, Sigma) and gluconic acid assay (Cat. No. MAK279, Sigma) kits. To investigate the activity and stability of free-state and immobilized GDH, BWH was used as the sole glucose source. The glucose concentration of the reaction mixture was set to 100 mM using BWH liquor. Free and immobilized GDH were tested in 10-mL reactions in 50 mL sterile Falcon tubes placed in an orbital shaker at 200 rpm and 55 °C. Samples were taken during 3-h reactions. Before measuring NADH, glucose and GA concentrations, samples were filtered through 0.2-μm filters and deproteinized using 10-kDa cut-off spin filters. As previously described, after 3 h the supernatant was removed and the supports with immobilized enzymes were kept in ES overnight at 4 °C and re-used in a new BWH-containing reaction mixture 10 times.

### SEM analysis

Scanning electron microscopy (SEM) images were taken using a JEOL JSM-7800F instrument equipped with EDX and operating at 10 kV. The samples were mounted using carbon tape on a sample holder. The images were also taken before and after enzyme immobilization to check the distribution of enzyme on the supports and to confirm their structural integrity.

### Statistical analysis

Data were reported as the mean of at least three experiments. Error bars denote standard deviations.

## Supplementary information

**Additional file 1: Table S1.** Effect of the storage period and the support material on GDH leakage.

## Data Availability

Data supporting our findings can be found in Additional file 1 which has been provided as additional material.

## Abbreviations

BCA: Bicinchoninic acid assay
BWH: Bread waste hydrolysate
EDX: Energy-dispersive X-ray analyser
ES: Enzyme support solution
GA: Gluconic acid
GDH: Glucose dehydrogenase
IPTG: Isopropyl ß-d-1-thiogalactopyranoside
MM-SBA-15: Hierarchically porous silica support
SDS: Sodium dodecyl sulfate
SDS-PAGE: Sodium dodecyl sulfate–polyacrylamide gel electrophoresis
SEM: Scanning electron microscopy
