# Supplementary material for: Purification and immobilization of engineered glucose dehydrogenase: a new approach to producing gluconic acid from breadwaste
Source: Biotechnol Biofuels. 2020 Jun 3;13:100. doi: 10.1186/s13068-020-01735-7 (PMC7268246; doi:10.1186/s13068-020-01735-7)
Supplement: Supplementary file 1 — Additional file 1: Table S1. Effect of the storage period and the support material on GDH leakage. [file 13068_2020_1735_MOESM1_ESM.docx]

Supplementary Table 1. Effect of the storage period and the support material on GDH leakage

| **Support Material** | **ECR8309F** | **ECR100M** | **ECR1030M** | **MM-SBA-15-300** | **MM-SBA-15-200** |
| --- | --- | --- | --- | --- | --- |
| Protein leakage (%) after 8-week storage | **24±7** | **31±1** | **36±3** | **35±1** | **36±1** |
| Protein leakage (%) after 12-week storage | **30±2** | **30±1** | **34±1** | **40±1** | **43±3** |
